# Diagnosis of Superficial Gastric Lesions Together with Six Gastric Lymphoma Cases via Probe-Based Confocal Laser Endomicroscopy: A Retrospective Observational Study

**DOI:** 10.1155/2018/5073182

**Published:** 2018-06-13

**Authors:** Qian Chen, Heng-Hui Cheng, Shuang Deng, Dong Kuang, Chang Shu, Li Cao, Guang-Quan Liao, Qiao-Zhen Guo, Qi Zhou

**Affiliations:** ^1^Endoscopic Unit, Department of Gastroenterology and Hepatology at Tongji Hospital, Tongji Medical College, Huazhong University of Science and Technology (HUST), Wuhan, China; ^2^Department of Pathology at Tongji Hospital, Tongji Medical College, HUST, Wuhan, China; ^3^Tongji Medical College, HUST, Wuhan, China; ^4^Department of Epidemiology and Biostatistics, School of Public Health, Tongji Medical College, HUST, Wuhan, China

## Abstract

**Objective:**

To evaluate the performance of probe-based confocal laser endomicroscopy (pCLE) in diagnosis of gastric lesions.

**Methods:**

An outpatient department- (OPD-) based retrospective study was conducted for patients with suspected upper gastrointestinal (GI) tract lesions who underwent pCLE between 2014 and 2016 at a tertiary hospital in China. Final diagnosis was based on the histopathological reports. CLE reports were compared to histopathological reports to evaluate the diagnostic ability, including sensitivity, specificity, positive predictive value (PPV), negative predictive value (NPV), and diagnostic accuracy.

**Results:**

322 of 380 patients were diagnosed with gastric lesions via pCLE, including inflammation and benign ulcers (*n* = 110), atrophy and intestinal metaplasia (*n* = 152), intraepithelial neoplasia (*n* = 27), adenocarcinoma (*n* = 27), and lymphoma (*n* = 6). In total, the diagnostic ability of CLE in evaluation of gastric lesions showed sensitivity 72.4% (95% confidence interval (CI): 67.1–77.2%); specificity 93.1% (95% CI: 5.6–8.4%); PPV 72.4% (95% CI: 67.1–77.2%); NPV 93.1% (95% CI: 5.6–8.4%); and accuracy 88.9% (95% CI: 87.3–90.4%), respectively. We further observed the capability of pCLE in diagnosing six gastric lymphoma showing those affected mucosa densely infiltrated with identical and round-shaped abnormal cells. Immunohistochemistry analysis confirmed one patient with diffuse large B-cell non-Hodgkin's lymphoma (DLBCL) and five with mucosa-associated lymphoid tissue (MALT) lymphoma.

**Conclusion:**

pCLE is an accurate tool for the detection of gastric lesions and shows optimal values of sensitivity and negative predictivity. Moreover, combining pCLE with white light endoscopy (WLE) may be a promising adjunct to conventional biopsy sampling in evaluating GI tract with suspected lymphoma.

## 1. Introduction

CLE technology is an emerging technology and enables endoscopists to collect real-time *in vivo* histological images or “virtual biopsies” of the gastrointestinal (GI) mucosa at high resolution [1]. With 1000x magnification of the mucosal layer, the epithelial cells, connective tissue, and changes in vascular patterns of GI tract can be assessed during endoscopy [2]. At present, the potential role of CLE in revealing premalignant and malignant lesions has been of extreme relevance to different pathologic conditions. Accordingly, many studies demonstrate a high correlation between CLE and histopathology results with accuracy ranging from 86% to 96% [3].

Two CLE-based systems are used in routine clinical practice and research. One is an endoscope-integrated CLE (eCLE) system that collects images at a manually adjustable scan rate of 1.6 frames per second. The optical slices of this specialized endoscope are parallel with the mucosal surface with a lateral resolution of 0.7 *μ*m, and the scanning depth can be dynamically adjusted from 0 to 250 *μ*m [2, 4]. In contrast to the eCLE system, pCLE has a fixed scanning depth of 55–65 *μ*m. Nevertheless, pCLE image data are collected at 12 frames per second, enabling real-time video quality and direct visualization of blood on a single erythrocyte scale [5]. Depending on the probe used, the field of view ranges from 240 *μ*m to 600 *μ*m, whereas fixed 475 *μ*m in diameter for eCLE [6]. Thus, the advantage of eCLE system is its high resolution whereas pCLE probe denotes greater flexibility to be introduced through the working channels of any kinds of endoscopes [3, 4, 6].

Currently, CLE technique is not yet routinely used in the clinical practice although it has the prospective impact to improve diagnosis and treatment approach for patients. Many factors, including the cost of the procedure, no clear indications for standard of care, and also the lack of image interpretation training for physician, limit its application in clinics. Furthermore, lack of cross talk between pathologist and physician but relying on histopathology for the final diagnosis of diseases could lead to a 20 to 30% misdiagnosis rate [3]. On the basis of these considerations, we retrospectively analyzed 380 patients who underwent OPD-based pCLE procedures from October 13, 2014, the first day when it became available at our hospital, to December 30, 2016. We aimed to evaluate its diagnostic accuracy and furthermore to validate the use of pCLE in managing selected diseases with diagnostic uncertainties, of which may result in the improvement of therapeutic decisions and/or follow-up procedures in these patients.

## 2. Materials and Methods

### 2.1. Single Endoscopy Unit Study

A database of all patients examined by pCLE procedure from October 13, 2014, to December 30, 2016, at the OPD-based endoscopy unit of Tongji Hospital, HUST, was accessed. Tongji Hospital is a state-owned teaching hospital. The endoscopy unit has availability of all endoscopic facilities and treatment modalities for diagnostic, therapeutic, and palliative endoscopies, including narrow band imaging (NBI) and pCLE examination. To detect and localize suspicious areas in GI tact, physicians at Tongji Hospital initially referred these patients for either NBI or pCLE examination. However, patients made final decisions after being informed between the two procedures, based on their financial status and also health insurance policy. In the study period, about 362 patients with 386 lesions chose pCLE at this unit, including 55 esophageal-cardia, 327 gastric, and 4 duodenal lesions.

### 2.2. Retrospective Analysis

For the purposes of this study, the records of the endoscopy unit were retrospectively reviewed, as well as hospital medical records. All authors had access to information that could identify individual participants during data collection. Approval for the study was given by the Institutional Ethics Committee.

### 2.3. CLE Criteria for Gastric Superficial Lesions

The pCLE criteria were based on the 2011 Miami classification [5] and Qilu classification for gastric superficial lesions [7–9]. Four pCLE diagnoses were given through evaluating architecture of glands, cells, and microvessels as follows: (1) normal gastric mucosa or benign inflammatory lesions were defined as regularly ranged glands with good polarity, and when inflammation occurred in gastric body, noncontinuous short rod-like glands with short thread-like opening could be seen or when inflammation occurred in antral mucosa, elongated and tortuous branch-like glands could be seen, with honeycomb-like microvessels (gastric body) or coil-shaped microvessels (gastric antrum); (2) atrophy and/or intestinal metaplasia (IM) was defined as the number of glands decreasing with dilating appearance for atrophy and uniform villiform architecture for IM cases, with characteristic black goblet seen in IM, with normal caliber, honeycomb-like or coil-shaped microvessels; (3) intraepithelial neoplasia (IEN) was defined as impaired gland polarity with irregularity in size and epithelial heights, with abnormal cell polarity and increased stratification and hyperdense epithelial cells, and with dilated and distorted microvessel appearance; (4) cancer was defined as the appearance of destroyed gland architecture and loss of gland polarity, absence of cell polarity with disordered appearance, and increased calibre microvessels with irregularity in size and shape, respectively, [5, 7–9].

CLE criteria for gastric lymphoma were based on recent established findings as follows [10–12]: (1) darkened areas made up of small roundish cells of similar size and morphology for the mucosa-associated lymphoid tissue (MALT) lymphoma or larger roundish cells for diffuse large B-cell lymphoma (DLBCL); abnormal cells in a dense arrangement; (2) cellular infiltrates typically affecting but not limited to the lamina propria; (3) cellular invasion of various epithelial structures; (4) altered tissue morphology to complete loss of structural integrity of the epithelium.

### 2.4. Endoscopy Equipment and Procedure

All procedures were performed using an Evis Lucera Spectrum system (Olympus Medical Systems Co., Tokyo, Japan) with a high-resolution upper GI zoom endoscopy (GIF-Q260, Olympus). After a mucosal lesion was visualized by WLE, fluorescein-aided pCLE was performed with the GastroFlex UHD miniprobe (Cellvizio; Mauna Kea Technologies, Paris, France). To obtain control pCLE images, the probe was first gently contacted to normal mucosa around the lesion, ideally showing regular round or oval glands with homogeneous epithelial cells. The probe was subsequently moved to suspicious lesion to obtain pCLE image, and following it, biopsies were obtained from the area. The diagnosing of gastric lymphoma was based on endoscopic appearance (varying from slight mucosal irregularities to large ulcers) and CLE findings followed by the histopathological examination of 8–10 biopsies taken from suspicious GI lesions [13]. The pCLE images used in the study were taken by two endoscopists (Q.C. and Q.Z.), each with at least a three-year experience in performing endoscopy procedures.

### 2.5. Histopathology Assessment for Gastric Lymphoma

The lymphomas were categorized in accordance with the 2008 World Health Organization classification of hemopoietic malignancies [14]. A histopathological diagnosis of MALT lymphoma was made when a Wotherspoon histologic score of any one specimen was 5, indicating the presence of dense, diffuse infiltrate of centrocyte-like cells in the lamina propria with prominent lymphoepithelial lesions [15]. In all patients of suspicious lymphoma, paraffin sections were processed with immunochemical techniques for the demonstration of light chain restriction and the phenotypes CD20, CD79, CD10, BCL-6, c-myc, MUM1, CD3, CD43, CD5, BCL-2, cyclin D1, Ki-67, CD21 and CD23, and so on. DLBCL is characterized by expression of B-cell-associated antigens (CD19, CD20, and CD79), with high-Ki-67 proliferation index [16]. Accordingly, BCL-6 expresses in approximately 80% of DLBCL and expression of CD10 and multiple myeloma oncogene (MUM-1) is associated with poor prognosis [17, 18]. The immunophenotype of a MALT lymphoma cell recapitulates that of the marginal zone B-cell. Typically, tumors express pan-B antigens (CD19, CD20, CD22, and CD79a), but they lack CD5, CD10, CD23, and BCL-1 expression. In rare cases, MALT lymphomas exhibit aberrant CD5 expression, which may be associated with a more aggressive clinical course [13, 19, 20].

### 2.6. Statistical Analysis

Categorical variables were summarized with number and percentage. The diagnostic accuracy of CLE and the prediction of gastric lesions in comparison to standard histology were assessed by using StatsDirect statistical software. Sensitivity and specificity were estimated for pCLE, along with exact bionormal 95% confidence intervals (95% CI), with standard histopathology diagnosis serving as gold standards. PPV = (number who have disease and screened positive) × 100%/[(number who have disease and screened positive) + (number who have no disease and screened positive)]. NPV = (number who have disease and screened negative) × 100%/[(number who have disease and screened negative) + (number who have no disease and screened negative)].

## 3. Results

### 3.1. Demographic Baseline Data

Between October 13, 2014, and December 30, 2016, a total of 327 gastric lesions from 327 patients using WLE and pCLE procedures were retrospectively assessed at OPD-based endoscopy unit in HUST. Five patients were excluded from the investigation due to missing histopathological diagnosis, and overall, 322 patients (225 males, 102 females) were analyzed. Thus, median age of patients at the time of pCLE was 53 years (range, 22–78 years) among 322 patients (Table 1). The final diagnosis was given based on standard histology results, leading to a diagnosis of benign inflammatory lesions such as gastritis or benign gastric ulcers in 110 cases (34.1%), atrophy and/or IM in 152 cases (47.2%), IEN in 27 cases (8.4%), adenocarcinoma in 27 cases (8.4%), and lymphoma in 6 cases (1.9%), respectively.

### 3.2. Diagnosis Accuracy of pCLE Compared to Histopathology

Final diagnosis was categorized based on pathology reports. Except the inflammation or benign ulcerative lesions, for those suspected with precancerous or malignant lesions, their individual-linked IHC reports were extracted to confirm the diagnosis. pCLE images in combination of WLE revealed abnormalities that led to a suspicion of inflammation or benign ulcer in 45 lesions compared to 110 diagnosed by histopathology; a suspicion of 132 atrophy and/or IM among 152 lesions; and 26 IEN among 27 lesions, 24 cancer among 27 lesions, and 6 lymphoma, respectively. In total, 233 pCLE findings matched the histopathology results. Thus, the sensitivity, specificity, PPV, NPV, and accuracy in diagnosing inflammation or benign ulcer compared to histopathology (*n* = 110) were 40.9%, 94.8%, 80.4%, 75.6%, and 76.4%, respectively. The sensitivity, specificity, PPV, NPV, and accuracy for diagnosing atrophy and/or IM were 86.8%, 81.8%, 81%, 87.4%, and 84.2%; 96.3%, 87.1%, 40.6%, 99.6%, and 87.9% for diagnosing IEN; and 88.9%, 97%, 72.7%, 99%, and 96.3% for diagnosing cancer, respectively (Table 2). In addition, six lymphoma cases were able to be correctively diagnosed following CLE criteria [10–12]. Together, pCLE showed sensitivity of 72.4% (95% CI: 67.1–77.2%), specificity of 93.1% (95% CI: 5.6–8.4%), PPV of 72.4% (95% CI: 67.1–77.2%), NPV of 93.1% (95% CI: 5.6–8.4%), and diagnostic accuracy of 88.9% (95% CI: 87.3–90.4%), respectively (Table 2).

### 3.3. pCLE in Diagnosing Gastric Lymphoma Compared to Histopathology

**Figure 1:**
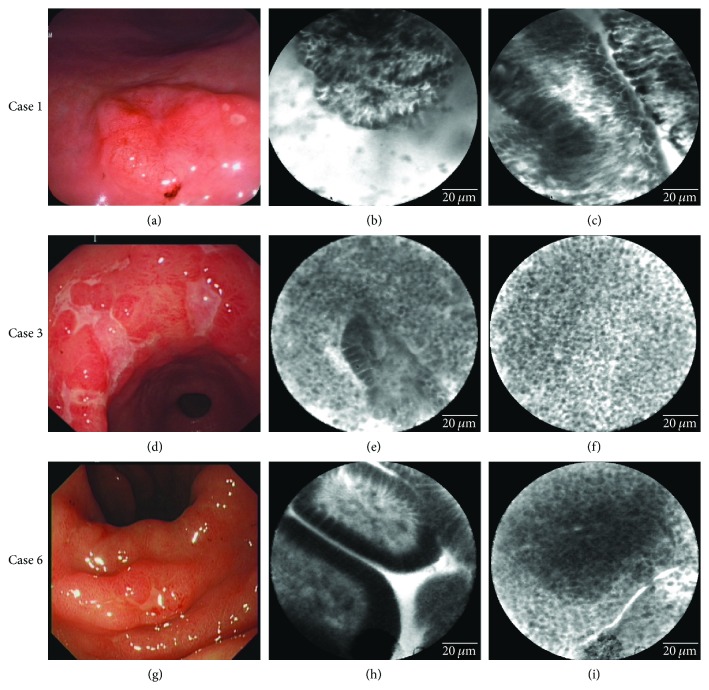
WLE and pCLE imaging of gastric lymphoma for case 1 (a–c, DLBCL), case 3 (d–f, MALT lymphoma), and case 6 (g–i, MALT lymphoma). The conventional endoscopic imaging revealed a single-nodule lesion with depressed surface in gastric body (a), multiple ulcerative lesions in gastric angular and antrum (d), and erosions and erythematous lesions in gastric angular (g). In the DLBCL case, pCLE revealed many dark and large roundish cells infiltrated widely in the exiting glands in a sheet-like fashion (b, c). In a MALT lymphoma case, pCLE revealed a massive infiltrate of small roundish cells with similar size and morphology, and in a dense arrangement (f, i), lymphoepithelial lesions could be observed (e, f, i). In (e), most pylorus glands were replaced by lymphocytic infiltrate except one gland showing the impaired structure infiltrated with small and roundish cells. In contrast, normal angular glands in the mucosa around the lesion in case 6 (h) showed regular oval or short rod-like pit pattern with homogenous epithelial cells.

In our retrospective study, we reviewed the pCLE imaging of those lymphomas (Table 3) and observed the affected mucosa infiltrated with densely identical and round-shaped abnormal cells (Figure 1). In DLBCL case, pCLE revealed many dark and large roundish cells infiltrated widely in the exiting glands in a sheet-like fashion (Figures 1(b) and 1(c)). In MALT lymphoma, pCLE revealed lymphoepithelial lesions (Figure 1(e)) and massive infiltrate of small roundish cells with similar size and morphology in a dense arrangement (Figures 1(f) and 1(i)).

**Figure 2:**
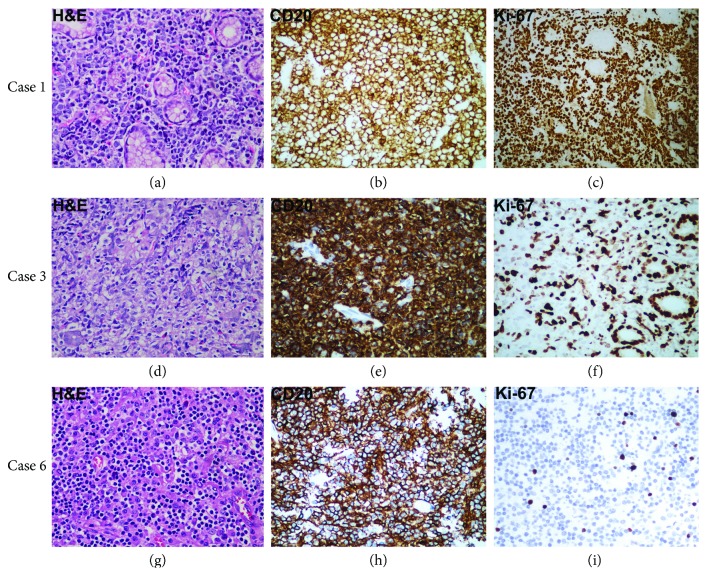
Histopathological examination for case 1 (a–c, DLBCL), case 3 (d–f, MALT lymphoma), and case 6 (g–i, MALT lymphoma). For DLBCL, hematoxylin and eosin (H&E) staining shows massive large cell infiltration with vesicular nuclei, prominent nucleoli, and basophilic cytoplasm (a, ×40). For MALT lymphoma, H&E staining shows dense diffuse infiltrate of centrocyte-like cells and the presence of lymphoepithelial lesions (d, g; ×40). Immunohistochemistry (IHC) sections showing infiltrating lymphoma cells express strong positivity with anti-CD20 stain, confirming their B-cell origin (b, e, h; ×400). (c, f, i) indicate the IHC staining for Ki-67 (×400). Positive staining is indicated by the brown color. In the DLBCL case (c), the neoplastic cells show prominent immunoreactivity to Ki-67.

Histology staining further showed massive large cell infiltration with vesicular nuclei, prominent nucleoli, and basophilic cytoplasm in DLBCL (Figure 2(a)), and dense diffuse infiltrate of centrocyte-like cells and the presence of lymphoepithelial lesions in MALT lymphoma (Figures 2(d) and 2(g)). IHC analysis confirmed that tumors express pan-B antigens (CD20, CD79), but lack CD5 expression (Table 4 and Figure 2). DLBCL was focally positive for BCL-6, with high Ki-67 proliferation index. In contrast, the five MALTs were BCL-6^−^ and CD10^−^, indicating no transformation of follicular lymphoma occurred. Except case 6, MALT lymphomas expressed MUM-1, indicating its origin of postgerminal center B-cells, but may be associated with a more aggressive clinical course (Table 4) [17, 18].

## 4. Discussion

The present study retrospectively evaluated patients who have been performed pCLE procedures based on a single-center experience. We demonstrated that a pCLE system could be a useful tool to examine patients with gastric lesions (*n* = 322) with sensitivity of 72.4%, specificity of 93.1%, PPV of 72.4%, NPV of 93.1%, and diagnostic accuracy of 88.9%, respectively (Table 2). Accordingly, many studies have demonstrated a high correlation between CLE and histopathology results with accuracy ranging from 86% to 96% [3]. In particular, several studies indicate that the pCLE system has an advantage in predicting IEN with a high level of accuracy of 99%, 97.4% of sensitivity, and 97.4% of specificity [21]. However, we show that the sensitivity, specificity, PPV, NPV, and accuracy in diagnosing IEN compared to histopathology (*n* = 27) were 96.3%, 87.1%, 40.6%, 99.6%, and 87.9% (Table 2); it is currently difficult to make direct comparisons of our study to others. It is possible that our group with less experience is still “on the learning curve.” Further study on the learning curve of pCLE imaging is warranted and underway.

Gastrointestinal lymphoma is the most common form of primary extranodal non-Hodgkin's lymphoma (PE-NHL) referring to lymphomas originated from any organ or tissue other than lymph nodes or spleen [13]. For gastric NHL, the majority is B-cell lymphoma with two main histological subtypes (>90% of cases), including MALT lymphoma and DLBCL [13, 22, 23]. Currently, the diagnostic approach for patients with gastric lymphoma is based on thorough upper gastrointestinal endoscopy [24]. When gastric lymphoma is suspected, the most frequent problem in the diagnosis is its differentiation from *H. pylori*-associated gastritis. Particularly at OPD settings, those cases arise in patients in whom standard WLE showed nonspecific macroscopic features such as inflammation, thicken folds, superficial erosions, or ulceration [25]. The histological features favoring MALT lymphoma have been previously reported, including a dense lymphoid infiltrate dominating most of the biopsy specimens, prominent lymphoepithelial lesions, Dutcher bodies in plasma cells, and infiltration of muscularis mucosae and centrocyte-like cells, which are small to medium-sized cells with small irregular nuclei [19, 20]. For DLBCL, lesions are characterized by an intense cellular infiltration of the lamina propria and the predominant cells resemble either centroblasts (large noncleaved cells) or immunoblasts [16]. Nevertheless, many endoscopic biopsy specimens lack several of these features. Therefore, at present, repeated biopsy and pathology play important roles in diagnosis and management of the diseases. Occasionally, gastric lymphoma can present as a multifocal stomach disease with numerous clonally identical foci in macroscopically unaffected tissue [13]. Overall, extensive biopsy sampling and gastric mapping of those macroscopically unaffected mucosa are crucially recommended in order to establish diagnosis. In terms of determination of the depth of invasion and the sensitive detection of affected regional lymph nodes, endoscopic ultrasonography (EUS) has the great impact in locoregional staging of the disease [24].

Our study confirms two previous findings, which demonstrate the applicability of CLE in diagnosing of gastric lymphoma [11, 12]. The first study by Dolak and colleagues used a pCLE system with a fixed scanning depth of 55–65 *μ*m and characterized that a dense infiltration of small cells with a size of approximately 5 *μ*m was a special finding for gastric MALT lymphoma [11]. Recently, a pilot study by Nonaka and colleagues set up descriptive criteria for MALT lymphoma by using integrated CLE, which in contrast to pCLE is a confocal scanner integrated into the distal tip of endoscope and the scanning depth can be dynamically adjusted from 0 to 250 *μ*m [12]. The evaluation of 24 patients with gastrointestinal MALT lymphoma by comparing EUS, WLE, and CLE suggested that their sensitivity was 80%, 100%, and 93%, whereas the specificity was 67%, 23%, and 100%, respectively. It is also highlighted that identification of darkened areas made up of small roundish cells of similar size and morphology is the criteria together with the cellular infiltration patterns as well as altered tissue morphology of which are actually quite close to conventional histology and immunohistochemistry findings. Although pCLE has a fixed scanning depth of 55–65 *μ*m, in clinical practice, we are able to visualize varying depths of mucosa, including the lamina propria by increasing or decreasing the pressure of the probe against the GI wall. Thus, those infiltrated lymphoid cells and lymphoepithelial lesions, we observed by pCLE lesions in lamina propria and epithelium. Accordingly, our current study confirms the effectiveness of pCLE in diagnosing lymphoma and thus suggests that CLE may serve as a promising adjunct to conventional biopsy sampling. However, the limited sample size is regarded as a shortcoming, and thus, a controlled, blinded prospective clinical trial should be initiated in a large cohort in the future study.

Till now, the characteristics of DLBCL have not clearly been addressed by CLE approach, although several reports have valued CLE in diagnosing DLBCL [10, 26]. This may be due to the fact that such deep diagnostic tools have not been routinely used in GI lymphoma staging and follow-up. At our endoscopy unit, we offer patient options including EUS and narrow-band imaging in combination with magnification endoscopy (NBI-ME), as well as pCLE when lesions were suspected and required further assessment. Many patients preferred NBI-ME over pCLE because of lack of sufficient health care reimbursement. Previous studies have showed the application of NBI-ME in diagnosis of gastric lymphoma through observing the destroyed glandular structures and presence of branching abnormal blood vessels [25, 27–29]. Nevertheless, the absence of gastric pit pattern or a nonstructural mucosal pattern is frequently appeared in gastric cancer, and therefore, the distinction between gastric cancer and lymphomas might be difficult by magnifying endoscopy alone in certain cases. In our retrospective study, we showed that pCLE revealed DLBCL with many dark and large roundish cells infiltrated widely in the exiting glands and this finding served as the major feature. However, given that for example, those abnormal cells in a sheet-like fashion (Figures 1(b) and 1(c)) could also be shared by gastric cancer; biopsy is needed at present to distinguish them. One of the limitations of the current study was that the pathology or IHC data was from the previous horizontal-sectional histologic examination. Given that pCLE provides the cross-sectional virtual histologic images *in vivo* are obviously different from those found by horizontal pathological examination [7], in the current retrospective study, the relation between pCLE scanning depth and resultant pathological findings could not be addressed. Applying the cross-sectional pathological examination demonstrated by Zhang et al. [7] will be guaranteed in future study in order to further understand pCLE images. Another limitation of this study was those patients were OPD-based, and we could not check the rearrangement of BCL-2, BCL-6, or MYC gene arrangement to further confirm our diagnosis. Thus, in the future, more DLBCL patients should be monitored under pCLE system and followed up at our endoscopy unit in order to develop diagnostic criteria. It may help us in managing selected diseases with diagnostic uncertainties, of which may result in the improvement of therapeutic decisions and/or follow-up procedures in these patients.

In summary, the prediction of gastric lesions based on pCLE has a relatively high diagnostic accuracy as compared to histology analysis. Regarding gastric lymphoma, this novel technique maps the affected mucosa and aids biopsy sampling during endoscopic procedure and thus may reduce the number of unnecessary biopsy in order to establish diagnosis. Given that combining pCLE with WLE may be a promising alternative to evaluate GI tract with suspected lymphoma, new clinical trials on pCLE are warranted and ongoing.

## Figures and Tables

**Table 1:** Demographic characteristic of 322 patients with gastric lesions.

Variable	Summary (*n* = 322)
^∗^Age, Y	53 (22, 78)
Gender, *n* (%)	
Male	223 (69.3)
Female	99 (30.7)
Histopathology diagnosis	
Inflammation or benign ulcer, *n* (%)	110 (34.1)
Atrophy and/or IM, *n* (%)	152 (47.2)
IEN, *n* (%)	27 (8.4)
Adenocarcinoma, *n* (%)	27 (8.4)
Lymphoma, *n* (%)	6 (1.9)

^∗^Age was summarized as median (minimum and maximum). IM: intestinal metaplasia; IEN: intraepithelial neoplasia.

**Table 2:** Sensitivity, specificity, PPV, NPV, and diagnostic accuracy of pCLE for gastric lesions (*n* = 322).

	Inflammation or benign ulcer (*n* = 110)	Atrophy and/or IM (*n* = 152)	IEN (*n* = 27)	Adenocarcinoma (*n* = 27)	Lymphoma (*n* = 6)	Total (*n* = 322)
Sensitivity (%) (95% CI)	40.9 (31.6–50.7%)	86.8 (80.4–91.8%)	96.3 (81.0–99.9%)	88.9 (70.8–97.7%)	100 (54.1–100%)	72.4 (67.1–77.2%)
Specificity (%) (95% CI)	94.8 (2.6–9.1%)	81.8 (12.7–24.9%)	87.1 (9.3–17.3%)	97.0 (1.4–5.7%)	100 (98.8–100%)	93.1 (5.6–8.4%)
PPV (%) (95% CI)	80.4 (67.6–89.8%)	81.0 (74.1–86.7%)	40.6 (28.5–53.6%)	72.7 (54.5–86.7%)	100 (54.1–100%)	72.4 (67.1–77.2%)
NPV (%) (95% CI)	75.6 (19.4–30.1%)	87.4 (7.9–18.8%)	99.6 (0.1–2.1%)	99.0 (0.2–3%)	100 (98.8–100%)	93.1 (5.6–8.4%)
Accuracy (%) (95% CI)	76.4 (71.4–80.9%)	84.2 (79.7–88.0%)	87.9 (83.9–91.2%)	96.3 (94.0–98.1%)	100 (98.9–100%)	88.9 (87.3–90.4%)

CI: confidential interval; PPV: positive predictive value; NPV: negative predictive value; IM: intestinal metaplasia; IEN: intraepithelial neoplasia.

**Table 3:** Gastric lymphoma patients' clinical characteristics.

Case number	Age (Y)	Gender	Lymphoma	Macroscopic findings	Tumor location
1	56	Male	DLBCL	Single nodule with depressed surface	Body
2	49	Male	MALT lymphoma	Fold thickening and erosions	Body
3	50	Female	MALT lymphoma	Ulcers	Angular and antrum
4	20	Female	MALT lymphoma	Ulcers	Body
5	63	Male	MALT lymphoma	Fold thickening and erosions	Body
6	26	Male	MALT lymphoma	Erosions and erythema	Angular and antrum

MALT: mucosa-associated lymphoid tissue; DLBCL: diffuse large B-cell lymphoma.

**Table 4:** Immunohistochemistry features for the 6 gastric lymphoma patients.

Case number	1	2	3	4	5	6
CD20	+	+	+	+	+	+
CD79*α*	+	+	+	+	+	+
PAX-5	+	+	+	+	+	+
CD10	+	−	−	−	−	−
BCL-6	+		−	−	−	−
c-myc	+	−	−	−	−	−
Kappa	+	−	(Weak) +	(Weak) +	−	(Weak) +
Lambda	+	−	+	(Weak) +	−	+
MUM1	−	−	−	−	−	+
CD3	−	−	−	−	−	−
CD43	−	−	+	−	+	−
CD5	−	−	−	−	−	−
BCL-2	−	N/A	+	−	+	+
Cyclin D1	−	−	−	−	−	−
^∗^Ki-67	>95%	7%	30%	2%	5%	15%
CD21	−	N/A	N/A	+	+	+
CD23	−	N/A	N/A	N/A	+	+
Histology grades	High	Low	Low	Low	Low	Low

∗ indicates Ki-67 labeling.
